# Implementation of a pediatric antibiotic stewardship intervention across a large integrated health system: protocol to optimize antibiotic selection and prescription duration for acute respiratory tract infections in children

**DOI:** 10.1186/s43058-026-00915-0

**Published:** 2026-04-09

**Authors:** Timothy R. Fowles, Payal K. Patel, Allan M. Seibert, Adam L. Hersh, Tom Belnap, Bridgett Hanna, Julia E. Szymczak, Heather T. Keenan, Bradley J. Barney, Rajendu Srivastava

**Affiliations:** 1 Healthcare Delivery Institute, Intermountain Health, 5026 South State Street, Murray, UT 84107 USA; 2 Division of Infectious Diseases, Intermountain Health, Murray, UT USA; 3 Department of Pediatrics, University of Utah, Salt Lake City, UT USA; 4 Enterprise Analytics Department, Intermountain Health, Murray, UT USA; 5 Clinical Excellence Department, Intermountain Health, Murray, UT USA; 6 Department of Internal Medicine, University of Utah, Salt Lake City, UT USA; 7 Utah Data Coordinating Center, University of Utah, Salt Lake City, UT USA

**Keywords:** Antibiotic Stewardship, Implementation Science, Acute Respiratory Tract Infections, Patient Centered Outcome Research

## Abstract

**Background:**

Appropriate antibiotic prescribing for routine childhood illnesses in ambulatory settings remains a major challenge especially at scale. The frequent overuse and misuse of antibiotics may lead to side effects in the children receiving them and contribute to the emergence of antibiotic-resistant bacteria. Substantial evidence suggests that adherence to established antibiotic prescribing guidelines curtails negative consequences without compromising treatment outcomes. However, adoption of guidelines remains stubbornly static. Implementation studies show specific strategies are effective at improving antibiotic stewardship (e.g., audit and feedback, electronic medical records [EMR] tools); however, there is limited evidence on how these strategies function when implemented across a large health system. As a large integrated health system, Intermountain Health (IH) is well-positioned for a scaled implementation of guideline-concordance antibiotic stewardship for acute respiratory tract infections (ARTIs) in children due to its previous stewardship work, culture of continuous quality improvement, and implementation infrastructure.

**Methods:**

This study will evaluate the implementation of guideline-concordance prescribing for Acute Respiratory Tract Infections (ARTIs) in children 6 months to less than 18 years of age in ~ 250 sites across 5 care specialties (pediatrics, family medicine, urgent care, emergency department, and telehealth). The evaluation will focus on: (1) the overall effectiveness of implementation strategies at driving adherence to guideline-concordant care across the organization, (2) the nature of variability in strategy implementation and adherence by context (e.g., specialty, rurality, region), and (3) the utility of a more intensive implementation, termed “Boost,” for sites with lower adherence after general implementation. Primary effectiveness outcome is adherence to guideline-concordant antibiotic *selection.* Secondary outcomes include adherence to guideline-concordant antibiotic prescription *duration*, and the combination of *both selection and duration* termed Recommended Antibiotic and Duration Adherence Rate (RADAR). We will employ an observational, quasi-experimental approach comparing adherence before and after implementation in two waves across the organization and pre-post Boost for sites selected for intensive implementation support. In addition, we will investigate the correlations between use of implementation strategies and changes in adherence.

**Discussion:**

This study aims to fill a critical gap in the literature concerning implementation of antibiotic stewardship at large scale. Following established implementation science regarding effective implementation strategies, this evaluation tests their utility across diverse settings thus enabling investigation of contextual determinants. In addition, the evaluation will investigate the response to general implementation strategies at scale as well as more targeted strategies, akin to academic detailing, for selected sites which require additional focused resources.

**Trial registration:**

ClinicalTrials.gov registration #NCT07334795.

**Supplementary Information:**

The online version contains supplementary material available at 10.1186/s43058-026-00915-0.

Contributions to the literature

- This study addresses a literature gap concerning implementation of antibiotic stewardship *at large scale* (~ 250 sites) and will provide key learnings about how integrated health systems can implement outpatient stewardship as a measurement-driven implementation program, leveraging a shared EHR, standardized dashboards, and routine feedback loops to support adoption *and* sustainment.
- It tests the established use of evidence-based implementation strategies (e.g., audit & feedback) at scale by investigating the use of phased intensification; specifically, employing an initial phase with minimal site-level focus followed by more intensive facilitation (termed “Boost”; i.e., academic detailing) for sites needing more specific support and will provide implementation science learnings about the potential benefit of enhanced support for stewardship programs: not only whether “Boost” improves outcomes, but which settings and contexts respond to intensification and what non-response implies for future strategy selection.
- It is among the largest U.S. efforts to improve pediatric outpatient antibiotic prescribing in ambulatory settings and will provide insights for stewardship programs that must balance focused efforts and efficiency at scale, avoiding a one-size-fits-all implementation but still maintaining an enterprise-wide implementation strategy.
- Findings will offer a transferable approach for embedding evaluation into routine operations, supporting the broader goal of generalizable scale-up methods for outpatient stewardship across large, dispersed health systems.
- Our experience will provide insights about what strategies can be standardized and what approaches might benefit from a more tailored approach when disseminating outpatient stewardship programs across multiple specialties and regions within a single Enterprise.

## Background

### Ambulatory antibiotic use, overuse, and misuse in children

Studies have demonstrated that antibiotics are overused – some estimate that up to one-third of antibiotics prescribed in the US in outpatient settings may be unnecessary [1–3]. In childhood, antibiotic use is nearly universal, and inappropriate prescribing is a well-recognized concern. In a sample of 759 children in the US with private medical insurance, Kissler and colleagues found that by 5 years of age, children had received a mean of 6.8 antibiotic courses and 91% had received at least one course [4]. Most antibiotics are prescribed in outpatient settings and respiratory conditions account for > 70% of these prescriptions for children [5]. For example, acute otitis media (AOM) is the single most common reason for antibiotics in children, resulting in an estimated 5 million affected children and 10 million antibiotic prescriptions annually in the United States (US) [1, 5, 6]. Even when an antibiotic is indicated, it may be misused if the *choice* of agent and *duration *deviate from evidence-based and professional society recommendations [7, 8]. Broad-spectrum antibiotics are frequently used in place of recommended narrow-spectrum options, and courses longer than the recommended duration are commonly prescribed [9–13].

These patterns of overuse and misuse have significant consequences. Unnecessary antibiotic exposure promotes the emergence of antibiotic-resistant organisms [14]. Antibiotics also carry direct risks for pediatric patients: they disrupt the normal microbiome and have been linked to increased risk of certain chronic conditions [15–18]. Emerging data also suggest the burden of antimicrobial exposure in early life can impair the immunologic response to immunization [19, 20] and diminish vaccine efficacy. Additionally, a notable proportion of children experience adverse drug events from antibiotics including diarrhea, rash, and rarely, severe allergic reactions or *Clostridioides difficile *infection [11, 21]. Reported rates of these adverse drug-related events may underestimate the actual frequency of these reactions and side effects since many may not be reported by a child's caregiver or result in contact with the medical system. In addition to these more apparent consequences of antibiotic overuse and misuse there are also direct (e.g. more healthcare visits due to side effects) and delayed indirect economic impacts (e.g. lost school and workdays) that have significant societal burden. Thus, reducing inappropriate antibiotic use in children is a public health imperative to improve the acute and long-term outcomes for pediatric patients as well as minimizing unnecessary costs for health systems and society.

To address these concerns and with increasing recognition of the need to promote judicious antibiotic use in ambulatory care, the US Centers for Disease Control and Prevention (CDC) released the Core Elements of Outpatient Antibiotic Stewardship in 2016 with an expected update to be released in 2026 emphasizing how health systems can contribute to ambulatory antibiotic stewardship [22]. This framework provides strategies to achieve improvements in ambulatory antibiotic prescribing based on four core elements: commitment, action for policy and practice, tracking and reporting, and education and expertise. A variety of interventions using elements from the CDC framework have been implemented and studied to decrease unnecessarily prescribed antibiotics [23–25] and to optimize antibiotic selection and duration when antibiotics are prescribed [26–28]. However, prescriptions that are unnecessary, overly broad-spectrum, and treatment courses longer than the recommended duration persist and are common [29–31].

Despite literature suggesting improvements in appropriate antibiotic prescribing (right drug, dose, and duration when antibiotics are truly needed) are achievable and yield better patient outcomes, effective and sustained implementation remains elusive. Shifting threats to adherence, including those internal to a system or environment as well as external factors (e.g. case mix changes) often undermine gains from previous implementations. Therefore, we aim to utilize the following strategies in combination to enact large-scale, sustainable implementation: 1) audit and feedback, 2) clinical decision support tools in the EMR to guide clinicians at the point of care to recommended narrow-spectrum antibiotics for the recommended duration, 3) clinician champions and local leaders to influence peers and sustain focus on stewardship goals, 4) educational outreach with updated and regularly renewed system guidelines and resources for each respiratory condition and peer stewardship partners to provide personalized training and problem-solving with clinicians to address knowledge gaps, concerns, and misconceptions, and 5) patient education and engagement via brochures, shared-decision making aids, or other approaches to help clarify caregiver expectations about antibiotics when appropriate, their role, and symptomatic management. Each of these strategies has shown promise in controlled or smaller-scale studies. The challenge remains how to integrate and scale all of these tactics in a unified ambulatory stewardship intervention across the varied care delivery environments of a geographically dispersed large health system that realizes and sustains improvements in antibiotic prescribing.

### Intermountain Health

Intermountain Health (IH) is a large, not-for-profit integrated health system in the Mountain West organized into three major geographic regions: Canyons (Central and Northern Utah, Idaho, and Western Wyoming), Peaks (Colorado, Eastern Wyoming, Montana, New Mexico), and Desert (Nevada, Arizona, & Southwest Utah). Each region includes urban, suburban, and rural communities (see Fig. 3 in Supplementary Information). This integrated structure with 33 hospitals and nearly 400 clinics has enabled IH to pursue ambitious system-wide quality improvement initiatives [32–36]. These attributes create a conducive environment for large-scale implementation of best practices, such as antibiotic stewardship, with support from leadership and alignment with system priorities. Prior and ongoing ambulatory antibiotic stewardship efforts in our system (see Table S2 and Fig. S4) demonstrated reductions in antibiotic prescribing for respiratory conditions in urgent care (UC) [23] and identified opportunities for improving prescription durations for common infectious conditions in primary care (PC) [29]. Other ongoing efforts seek to understand optimal strategies to increase the uptake of watchful waiting for pediatric ear infections [37, 38] and sustaining improvements in respiratory antibiotic prescribing in UC.

Despite these successes, prior outpatient stewardship activities at IH have been siloed – confined within particular service lines (UC or PC only, for example) or specific regions. A more coordinated and unified system-wide approach is needed to achieve substantial improvements in pediatric antibiotic prescribing, sustain them, and create a durable ambulatory antibiotic stewardship initiative in perpetuity that can adapt to external pressures as well as patient, provider, and pathogen changes along with evolving evidence.

**Fig. 1:**
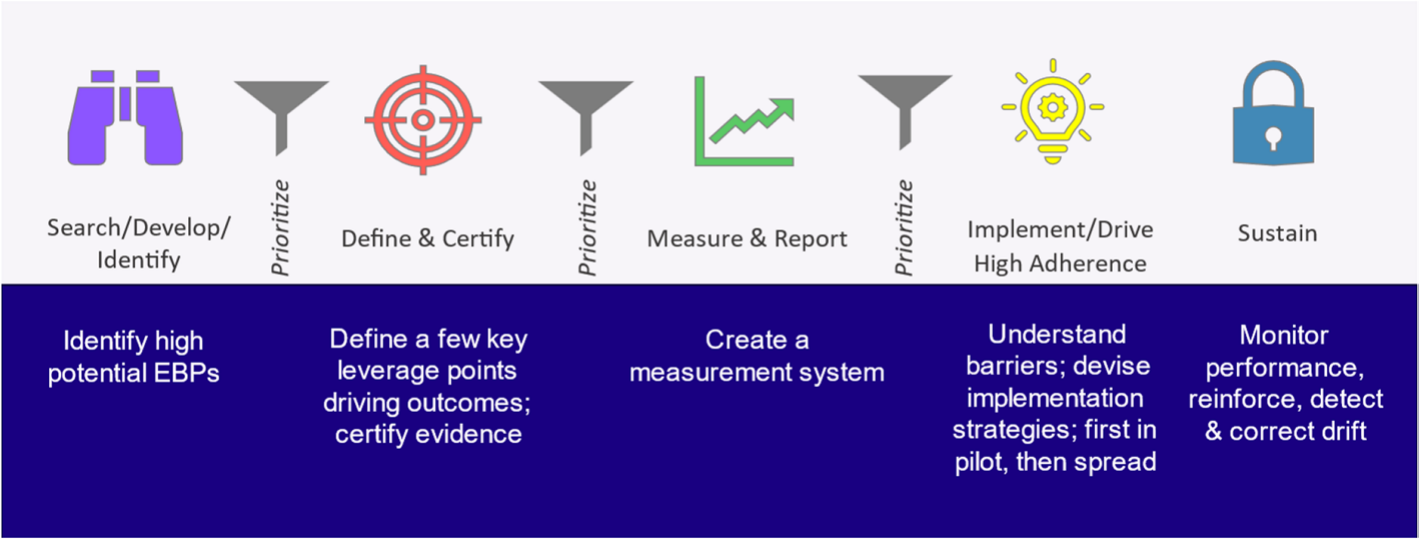
The Clinical Best Practice Integration (cBPI) model [39]

In addition to its history of antibiotic stewardship, IH is well-positioned to undertake this project based on the following unique characteristics:

1. A unified electronic health record (Epic) across all ambulatory and inpatient sites
2. An established culture of best-practice implementation and continuous quality improvement
3. System-wide infrastructure for implementing at scale including the Healthcare Delivery Institute, the Clinical Programs, and the Clinical Best Practice Integration (cBPI) model (see Fig. 1).

Each of these factors will be leveraged for the current project. EMR tools will be implemented across the system and trainings on the use of these tools rolled out with each implementation. The Healthcare Delivery Institute will manage the project and Clinical Programs are already working in lock-step to implement evidence based practice [40] and will leverage this relationship to implement and scale evidence-based stewardship for this project. The cBPI model [39] provides a clear framework already in use across IH that leverages and simplifies relevant implementation and improvement science tools to maximize the success of the stewardship project.

In combination, these IH characteristics (size, integrated EMR, culture of implementation/improvement, Healthcare Delivery Institute, Clinical Programs, cBPI) position IH to successfully implement a novel, unifying, outpatient antibiotic stewardship initiative at unprecedented scale. Additionally, our size and diversity provide singular opportunities to examine how context influences implementation. Within the ~ 250 care sites that will participate in this project there is substantial heterogeneity – from small rural clinics to large urban academic practices, general family medicine clinics to pediatric offices and telehealth, and a range of patient demographic groups. This heterogeneity across a broad geographic area of the US will enhance the generalizability of lessons learned. The integration of these sites within one system provides a common infrastructure that can be leveraged to deploy a standardized intervention and comprehensively assess its impacts across Intermountain and within regions.

In summary, IH offers a unique combination of breadth and connectedness, enabling both large-scale change and rigorous, nuanced evaluation of the implemented intervention to effect that change at the system, region, and care setting levels. Herein, we describe our implementation aims and strategies to optimize antibiotic selection and prescription duration for acute respiratory tract infections in children.

## Methods and design

**Fig. 2:**
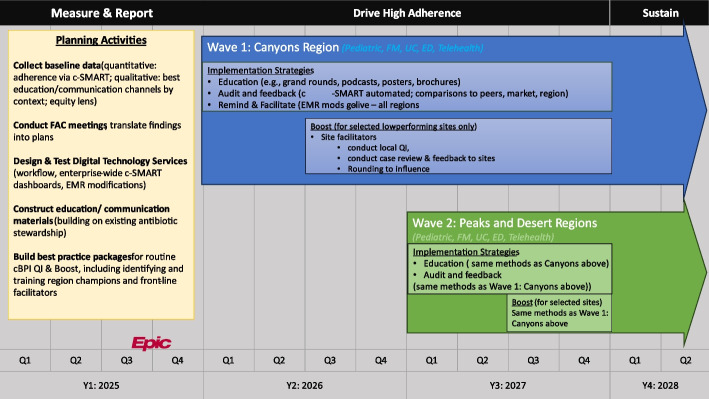
Phased implementation of SCORE-Peds

This project, named Stewardship in Community Outpatient settings – Resources and Engagement – Pediatrics (SCORE-Peds), leverages several key structures from Quality Improvement (QI) and Implementation Science (IS) in equal measure as described by Beidas et al. [41] The cBPI model is designed to leverage the strengths of QI and IS to integrate best practices across Intermountain. For example, the design and testing of digital technology solutions (e.g., EMR modifications) in the Measure & Report phase involves Plan-Do-Study-Act (PDSA) cycles to optimize the utility of these tools (Fig. 2). Likewise, sites will be selected for Boost using data on variation (QI). In response, QI teams will work at a local level to conduct site-specific root cause analyses and improvement cycles. In a complementary fashion the project employs specific implementation strategies from the Expert Recommendations for Implementing Change (ERIC) [42] established in IS studies of antibiotic stewardship. These include audit and feedback, clinician education, and facilitation (see Table 3 in supplementary information). We recognize that these approaches stem from separate traditions with differing approaches; however, like Beidas et al., we believe that a combined approach best serves the overall aims of this project as stated previously. Accordingly, we will reference both QI and IS approaches in the methods and design.

This initiative corresponds to the receipt of a Health Services Implementation Initiative (HSII) award [43] from the Patient-Centered Outcomes Research Institute (PCORI) in January of 2025. A foundation for this and similar initiatives was laid previously using a PCORI capacity building award under the HSII. Using capacity building funds, IH built a standardized dashboard system to use across all IH cBPI projects known as the cBPI Standardized Measurement and Reporting System (cSMART). In addition, specific to this project, the first two cBPI phases [39], Search/Develop/Identify and Define and Certify, were completed in preparation for the award. During the planning phase of SCORE-Peds, IH established workstreams to conduct key planning activities (see Fig. 2) corresponding to the measure and report phases of cBPI. During 2026 and 2027, IH will implement SCORE-Peds across all regions in all ambulatory settings where target conditions are treated. (Additional detail; see Table S3 for the Logic Model driving Quality Improvement [QI] and Table S4 for project activities).

### Study aims and hypotheses

#### Aim 1: Evaluate the overall effectiveness of core implementation strategies at driving high adherence to antibiotic guidelines for ARTIs across a large number of diverse clinical settings

##### Rationale

The project spans ~ 250 sites across the intermountain west (see Fig. S3) and multiple care settings (pediatrics, family medicine, urgent care, ED, telehealth) and offers a unique opportunity to evaluate the effectiveness of previously tested implementation strategies at scale.

##### Key questions

- Was adherence to antibiotic guidelines for ARTIs achieved (see Table 1 and Primary Innovation)?
- Did contextual factors (e.g., rural vs. urban, practice type, clinician role) influence adherence rates?
- Exploratory question: How did adherence change *over time* and did the time course differ by context?

##### Hypothesis

High adherence in each region will be achieved following implementation.

#### Aim 2: Identify how adherence varies by context and examine use of implementation strategies and contextual determinants

##### Rationale

The wide variety of geography and clinic types allows us to examine how contextual factors influence adherence, as well as use of implementation strategies while holding some things constant (e.g., shared EMR, corporate structure).

##### Key questions

- How do contextual factors (e.g., rural vs. urban, practice type, clinician role) influence adherence rates? How did that emerge over time and was the time course different in different contexts?
- What organizational, regional, and site-level factors facilitate or impede scale-up?
- Which implementation strategies are deemed most effective at driving high adherence based on contextual factors?
- Setting is key: traditionally, antibiotic stewardship has been siloed into specialties (e.g., pediatrics, family medicine, urgent care, ED). How does a large cross-setting initiative work?

##### Hypothesis

Use of implementation strategies (i.e., engagement) will correlate with changes in adherence.

#### Aim 3: Assess the impact of Boost on effectiveness and implementation outcomes

##### Rationale

The project includes an exploratory aim to test “Boost” – a more intensive implementation support program akin to academic detailing with sites that are less responsive to initial general implementation and therefore have lower rates of adoption. This allows for comparison of foundational vs. enhanced strategies and focused resource utilization.

##### Key questions

- Do academic detailing and similarly intensified strategies improve adoption?
- How do champions, clinical peer stewardship partners and site facilitators influence implementation climate and sustainment?

##### Hypothesis

Sites with lower adoption rates who receive the Boost will demonstrate increased use of implementation strategies and increased adherence compared to sites that did not receive Boost.

### Setting

The SCORE-Peds project spans a diverse set of care delivery environments across three major regions—Peaks, Canyons, and Desert—covering ~ 250 sites in the intermountain west (see Fig. S3). These settings include pediatrics clinics, family medicine clinics, urgent cares, emergency departments and telehealth. Site locations are also diverse and include urban areas (e.g., Salt Lake City, Utah; Denver, Colorado) as well as rural and suburban areas.

### Inclusion and exclusion criteria

Patients will include children aged 6 months to less than 18 years who were diagnosed with and treated for one of four common acute respiratory tract infections (ARTIs): acute otitis media, Group A Streptococcal (GAS) pharyngitis, acute sinusitis, or pneumonia. Children whose records include penicillin allergies will be excluded from evaluation.

### Study procedures

This evaluation will use observational and quasi-experimental designs to examine effectiveness and implementation outcomes as outlined below. The implementation will proceed in two waves with approximately half of the sites in each wave. To maximize efficiency and likelihood of adoption, sites will be assigned to waves based on region rather than by randomization (see Fig. 2). The first wave includes all sites in the Canyons region with Peaks and Desert regions in the second wave. Each wave will provide a pre-post design using archival baseline data. In addition, the Peaks and Desert regions serve as a comparison group for the Canyons implementation to compensate for possible secular trends. We plan to examine adherence rates overall (aim 1), across sites (aim 2), and as a function of response to Boost (aim 3).

In addition, we plan to examine the use of implementation strategies overall (aim 1), across sites (aim 2), and in response to Boost (aim 3). This examination is based on several key indicators including: the number and frequency of trainings, attendance at trainings, and the frequency with which providers and sites access tools such as EMR utilities (e.g., preference lists, order sets, smart sets), c-SMART dashboards, and audit & feedback reports. We expect that the use of these strategies will vary by site and correlate with changes in adherence.

### Data collection and analysis

Data collection will rely primarily on observational EMR data extracted from approximately 250 Intermountain Health sites, encompassing all pediatric encounters for indicated acute respiratory tract infections where antibiotics were prescribed during the study period. Baseline data from the 12 months prior to implementation will establish pre-intervention prescribing patterns, and ongoing EMR data abstraction will capture antibiotic selection, duration, and encounter-level covariates. Statistical analyses will follow a quasi-experimental pre–post design with staggered regional implementation, employing age, diagnosis, provider specialty, and care setting as covariates in linear probability models and using generalized estimating equations to account for clustering at the provider level. Clinic rurality and patient-level rurality, race, ethnicity, and preferred language may be controlled for if not deemed to be too collinear and not deemed as having too much missingness. The first three months of intervention will be considered an implementation rollout period and analyzed accordingly. Power calculations indicate > 99% power for a test at alpha = 0.05 to detect a ≥ 5% improvement in adherence across an estimated 356,000 encounters expected during the study period. If there is an overall effect, testing for each region separately will be conducted with Bonferroni-Holm adjustment for the three tests; if not significant, the region-specific tests will still be conducted but interpreted as exploratory.

Additional analyses will include interrupted time-series analysis and segmented regression to assess temporal trends, as in recent work [44]. Subgroup analyses will examine demographic covariates (e.g., ethnicity, language, insurance) and other characteristics (e.g., site rurality, setting type). Analyses will generally be conducted using robust variance estimators, unless explicit modeling of correlations is indicated (e.g., interrupted time-series analysis).

The Aim 3 analyses of Boost will proceed in an analogous manner, focusing on covariate-adjusted comparisons of adherence for encounters during the active implementation phase where Boost is in use to encounters during the active implementation phase where Boost is not in use.

### Primary innovation

Two complementary best practices will be tested: the primary outcome is adherence to recommended antibiotic *selection*.

Secondarily, we will examine adherence to recommended *duration*, as well as the combination of *selection and duration* (i.e., RADAR; see Fig. 5 in Supplementary Information). Prescriptions will be considered guideline-concordant if the antibiotic matches the recommendations in Table 1. Based on preparatory examination of data and charts, we will allow a 1-day window below or above the recommended duration (e.g., 4–6 days for 5 days).

**Table 1:** Recommendations for selection and duration based on ARTI condition

Condition	Age	Antibiotic	Duration
Acute Otitis Media	6 months—2 years old	Amoxicillin	10 days
	2–5 years old		5 days
	> 5 years old		5 days
Sinusitis	< 18 years old	Amoxicillin	5 days
Group A strep Pharyngitis	> 6 months old	Amoxicillin or Penicillin	10 days
Community Acquired Pneumonia	> 3 months	Amoxicillin	5 days

### Primary effectiveness and implementation outcomes

SCORE-Peds assigns value to both clinical effectiveness and implementation outcomes. Effectiveness outcomes will focus on improvements in guideline-concordant antibiotic prescribing for pediatric ARTIs.

The primary effectiveness outcome, which is the overall primary outcome with analyses as previously described, is the *antibiotic selection*– specifically the proportion of encounters in which a *narrow-spectrum antibiotic* is prescribed out of all ARTI encounters where any antibiotic was prescribed.

The primary implementation outcome will be two measures of *adoption* at the prescriber and site level. The measures of adoption are derivatives of the primary effectiveness outcome. Specifically, adoption is defined as the proportion of *prescribers* who have 80% adherence to antibiotic selection in their encounters, and the proportion of *sites* with 80% of prescribers considered adherent to antibiotic selection.

### Secondary effectiveness outcomes

An important secondary outcome is the proportion of encounters with an appropriate *duration* of antibiotic therapy out of all encounters where any antibiotic was prescribed. Appropriate duration is defined based on Table 1. The other secondary effectiveness outcome is the proportion of encounters with *both guideline-concordant selection and duration*. To facilitate consistent messaging with all stakeholders across our large health system, to describe this *R*ecommended *A*ntibiotic and *D*uration *A*dherence *R*ate we developed the acronym RADAR for this composite adherence measure (see Fig. 5 in Supplementary Information). This secondary effectiveness outcome is a more rigorous composite adherence measure that focuses on whether each antibiotic prescription for a target condition met both the recommended antibiotic selection and the recommended duration.

### Other outcomes

Treatment failure will be examined as a balancing measure and defined as a return visit between 2 and 14 days for the same ARTI diagnosis where a new antibiotic prescription was given.

In terms of implementation, the study will evaluate the proportion of sites using clinical decision support tools (e.g., preference lists for antibiotic selection and duration), the percentage of clinicians engaging with audit-and-feedback dashboards, and indicators of implementation engagement such as attendance at trainings, and participation in site facilitation.

### Data management

Study data will be collected from IH’s integrated electronic medical record (EMR) system and the corresponding IH enterprise data warehouse. These data will be used to report results to internal and external audiences supporting the ongoing QI. IH implemented a common EMR in September of 2025. Where available, baseline data from 2023–2025 will be collected to examine secular trends and inform the impact of the QI initiatives in 2026–2029. Data will include patient demographics, encounter data, medication and prescription information, area deprivation index (ADI) and other indicators of socio-economic status (e.g. RUCA codes), insurance data, care site characteristics (e.g., number of caregivers/prescribers at site, location of care site).

Evaluation of the project will include (1) the use of internal dashboards and reports to inform the QI process (i.e., formative evaluation), and (2) the use of robust statistical methods to evaluate the goals of the project (i.e., summative evaluation). For internal evaluation (1), Intermountain analysts will build dashboards and reports typical of quality improvement projects. For robust statistical analysis (2), the University of Utah Data Coordinating Center (DCC) will provide expert statistician support employing the analytic techniques described previously.

All data will be secured using established Intermountain policies for internal use and for the protection of research data. This includes oversight by the IRB at IH. Datasets used for advanced statistical analyses and summative evaluation will be protected as research data. This includes limiting access to evaluation personnel with appropriate research training, securing data when stored, and transmitting via encrypted channels.

## Discussion

Improving ambulatory antibiotic use in pediatrics remains a challenge and imperative for health systems. The SCORE-Peds project will provide significant insights for implementing antibiotic stewardship best practices at scale in a large geographically dispersed health system. To our knowledge, this is the first attempt to deploy a unified stewardship intervention across such a broad ambulatory network (250 sites) in real-world practice. This contrasts with prior stewardship studies which have been limited in scope – for example, focusing on a subset of clinics or a single region, or conducted as tightly controlled trials. By undertaking this system-wide approach, our study will yield practical learnings regarding logistics, effectiveness, differential impacts, and sustainability of scaling evidence-based antibiotic stewardship practices into routine care.

One anticipated benefit of operating at scale will be the ability to study contextual variation. With hundreds of sites involved and differences between each of our three distinct regions, we will be able to characterize how different settings respond to the same intervention with real-world experience. For instance, factors such as clinic size, rural vs urban patient population, care delivery setting, or baseline prescribing cultures may impact outcomes. We will be able to examine, for example, whether rural pediatric or family medicine clinics encounter different barriers to achieving high prescribing rates for narrow spectrum shorter duration prescriptions compared to large urban UC or ED clinics and how those barriers might be overcome. Such knowledge is highly valuable – large health systems often struggle with how to tailor improvement strategies to diverse practice environments. Our findings could inform guidance on what core components of an intervention need adaptation when spreading to new contexts versus what is more generalizable and can be standardized.

One notable innovation of this study is the use of the RADAR metric to track prescribing quality as a secondary effectiveness outcome. By linking the numerator to guideline-concordant prescriptions (recommended narrow spectrum antibiotic for the recommended duration) and the denominator to all encounters for the conditions of interest in which an antibiotic was prescribed, RADAR emphasizes adherence to clinical best practices at each opportunity. Traditional volume-based measures that simply count antibiotic prescriptions cannot distinguish recommended from inappropriate antibiotic use and may be less actionable in settings where overall prescribing rates are either already low or less amenable to interventions to decrease high prescribing rates. In contrast, RADAR directly highlights the appropriateness of therapy, aligning with stewardship goals of “right antibiotic, right duration” rather than simply decreasing prescription volume. There are however, unique considerations to RADAR. Clinical features such as case mix, severity, and prior history with antibiotics may not be easily captured. Like other metrics, RADAR may fail to fully capture important contextual nuances that can justify practices that deviate from guideline recommendations. It should be interpreted alongside clinical context, and qualitative feedback or case review may be needed to understand outliers. Nonetheless, the potential for RADAR to be used broadly in outpatient settings is significant, especially in health systems where prescription volume alone provides limited insight and our study will provide an opportunity to better understand its utility in a variety of ambulatory settings.

This study also includes a “Boost” phase wherein resources and efforts are directed to sites with lower adoption rates following an initial implementation. This design reflects an implementation intensification approach, augmenting low-touch tactics with high-touch support at sites in need of specialized support. Such strategies are increasingly recommended for scaling evidence-based practices in large health systems [45, 46]. Examining which contexts benefit the most from Boost will inform how to efficiently adapt and scale stewardship efforts across a large integrated health system. Importantly, if low-adoption sites do not improve with "Boost” efforts, this too will be an important learning and suggest that more intense application of certain stewardship strategies may not be the solution for sites and settings whose narrow-spectrum antibiotic prescribing rates for the recommended duration remain low. While our primary outcomes are focused on prescribing behavior, the ultimate goal is to improve patient health and experience, consistent with the Patient Centered Outcomes Research Institute’s (PCORI) mission. By reducing unnecessarily broad-spectrum antibiotic use and shortening antibiotic prescriptions when appropriate, we aim to decrease side effects and complications for children. We will indirectly assess patient-centered outcomes such as symptom resolution and satisfaction via treatment failure and leverage patient satisfaction survey data already a part of our health system standard patient experience to identify potential signals regarding antibiotic selection, duration, and RADAR.

### Challenges and limitations

This study serves as the evaluation of a large-scale implementation. Accordingly, it is not designed as a rigorous test of specific implementation hypotheses. While we intend to use robust statistical analyses and quasi-experimental approaches to investigate the stated aims, several aspects of the design limit the strength of causal inferences. For example, no randomization will be employed either for the implementation waves or for the selection of Boost sites. Given the scale, we are reliant on EMR data and clinician coding, which may introduce bias or misclassification.

We are focusing on process and proximal outcomes such as prescriptions for narrow-spectrum agents, prescriptions for shorter, recommended durations when appropriate, and the composite RADAR metric. We are not directly measuring long-term impacts such as changes in community antimicrobial resistance, microbiome alterations, or downstream health outcomes such as chronic health conditions (i.e. asthma, rheumatologic diseases, IBD) in patients who receive narrow vs broad-spectrum antibiotics or prescriptions for shorter durations. Our implementation goals are also vulnerable to unique seasonal or yearly case mix changes that can be challenging to predict (GAS 2022–2023, M pneumoniae 2024). Additionally, sustaining improvements post-intervention could be challenging once dedicated external project resources are withdrawn.

These limitations are deemed a necessary trade-off to accomplish our primary goal of developing and testing a large-scale implementation for pediatric ambulatory stewardship. To our knowledge, this is the first attempt to implement pediatric ambulatory stewardship across ~ 250 sites in one project. Thus, SCORE-Peds offers a unique opportunity to learn how to spread and maintain best practices across a large number and diverse array of outpatient settings. We will gain insights into which implementation strategies generalize across sites that share common systems and which require tailoring to address differences in case mix, geographic location, and practice type. This knowledge, coupled with the significant investment by PCORI and our focus on patient-centered outcomes, will inform future efforts to optimize ambulatory antibiotic use in pediatrics across integrated health systems on a broad scale and inform larger ambulatory antibiotic stewardship efforts.

## Supplementary Information

Supplementary Material 1.

Supplementary Material 2.

Supplementary Material 3.

Supplementary Material 4.

## Data Availability

Not applicable.

## Abbreviations

ADI: Area Deprivation Index
AOM: Acute Otitis Media
ARTI: Acute Respiratory Tract Infection
CDC: Centers for Disease Control and Prevention
CP: Clinical Programs
cBPI: Clinical Best Practice Integration
cSMART: cBPI Standardized Measurement and Reporting System
DCC: Data Coordinating Center
ED: Emergency Department
EMR: Electronic Medical Record
GAS: Group A Streptococcus
HDI: Healthcare Delivery Institute
HSII: Health Services Implementation Initiative
IH: Intermountain Health
IRB: Institutional Review Board
PC: Primary Care
PCORI: Patient-Centered Outcomes Research Institute
RADAR: Recommended Antibiotic and Duration Adherence Rate
RUCA: Rural-Urban Commuting Area
SCORE-Peds: Stewardship in Community Outpatient settings – Resources and Engagement – Pediatrics
UC: Urgent Care
