# Supplementary material for: Implementation of a pediatric antibiotic stewardship intervention across a large integrated health system: protocol to optimize antibiotic selection and prescription duration for acute respiratory tract infections in children
Source: Implement Sci Commun. 2026 Apr 9;7:93. doi: 10.1186/s43058-026-00915-0 (PMC13181975; doi:10.1186/s43058-026-00915-0)
Supplement: Supplementary file 1 — Supplementary Material 1. [file 43058_2026_915_MOESM1_ESM.pdf]

March 06, 2026

IRB # 1053614 **Study Alias:** SCORE-Peds
PI: Payal K Patel
Title: *Evaluation of antibiotic Stewardship in Community Outpatient settings – Resources and Engagement – Pediatrics (SCORE-Peds)*

Initial Application Exempt Determination
Acknowledged: 02/12/2026
Submission Reference #: 052267

The above-mentioned project has been reviewed by a member of the Intermountain IRB and it has been determined the project is exempt from 45 CFR 46 per category 45 CFR 46.104(d)(4).

If the scope of your project changes, please contact the Intermountain IRB immediately to ensure you do not need IRB approval or a Waiver of Authorization from the Privacy Board to continue with this project.

If you have any questions regarding this decision please contact the IRB analyst assigned to your study, Minsey Choi or call the IRB Office at (801) 408-1991 opt. 1.
