# Supplementary material for: Implementation of a pediatric antibiotic stewardship intervention across a large integrated health system: protocol to optimize antibiotic selection and prescription duration for acute respiratory tract infections in children
Source: Implement Sci Commun. 2026 Apr 9;7:93. doi: 10.1186/s43058-026-00915-0 (PMC13181975; doi:10.1186/s43058-026-00915-0)
Supplement: Supplementary file 2 — Supplementary Material 2. [file 43058_2026_915_MOESM2_ESM.pdf]

Letter of Award
PCORI Funding Announcement:
PCORI Health Systems Implementation Initiative (HSII)--Implementation Projects

Raj Srivastava
Intermountain Stewardship in Community Outpatient settings-Resources & Engagement (SCORE)-Pediatrics
IHC HEALTH SERVICES, INC.

9/25/2024

Dear Raj Srivastava,

Congratulations! On behalf of the Patient-Centered Outcomes Research Institute (PCORI), I am pleased to inform you that your application titled, "Intermountain Stewardship in Community Outpatient settings-Resources & Engagement (SCORE)-Pediatrics," has been approved for funding. We are pleased to be able to support your implementation efforts.

Please note the following:

1. PCORI awards contracts, not grants. This awarded Implementation Project will be incorporated as a Project Statement under your HSII Master Funding Agreement (MFA) contract. Even though your application has been approved, a final administrative and programmatic review must take place before you receive the award. PCORI will email you within several days to continue the award activation process. The Project Statement must be executed between your institution and PCORI before this funding is made available to you. Under the HSII MFA and Project Statement, the recipient accepts all terms and conditions as agreed upon by both parties. Until the Project Statement is fully executed, the proposed budget should not be construed as final. All pre-award costs are incurred by the applicant at risk.

2. Your summary statement is available via the following link:
https://pcori.my.salesforce.com/sfc/p/70000000Momb/a/UW000001vL8o/_RZVgBzngxQKKJ_ti6gLDY8DAWU.mmOjVXRnWIUjnB4
The summary statement contains assigned reviewers' written evaluations of your application.

3. EMBARGO NOTICE: PCORI will publicly issue details about the approved projects at 11:00 a.m. (EDT) on 10/2/2024. Your institution may communicate about this award publicly after 11:00 a.m. (EDT) that day.

4. If you wish to issue any public announcement related to this award, please coordinate with PCORI's Communications Department. You may contact PCORI Media Relations at mediarelations@pcori.org. Our Communications staff will reach out to you and share tools and resources (news release template and sample social media language) that may be useful.

Congratulations again on your selection to receive PCORI funding. We look forward to working with you to advance PCORI's mission. Please email us at HSIIQuestions@pcori.org if you have any questions.

Sincerely,

Nakela Cook, MD, MPH
Executive Director
Patient-Centered Outcomes Research Institute (PCORI)
1333 New Hampshire Avenue NW, Suite 1200
Washington, DC 20036
www.pcori.org | @PCORI

****Please note: Do not reply to this email. This email is sent from an unattended mailbox. Replies will not be read.****

© 2025 Patient – Centered Outcomes Research Institute. All Rights Reserved.
