# Supplementary material for: Implementation of a pediatric antibiotic stewardship intervention across a large integrated health system: protocol to optimize antibiotic selection and prescription duration for acute respiratory tract infections in children
Source: Implement Sci Commun. 2026 Apr 9;7:93. doi: 10.1186/s43058-026-00915-0 (PMC13181975; doi:10.1186/s43058-026-00915-0)
Supplement: Supplementary file 3 — Supplementary Material 3. [file 43058_2026_915_MOESM3_ESM.pdf]

Intermountain Health

March 06, 2026

Approval for Waiver of Authorization for Research by the Intermountain Healthcare Privacy Board

Date of approval: 02/12/2026
Name of Study: *Evaluation of antibiotic Stewardship in Community Outpatient settings – Resources and Engagement – Pediatrics (SCORE-Peds)*
Principal Investigator: Payal K Patel
IRB Number: 1053614

The Intermountain Healthcare Privacy Board has determined that the waiver of authorization satisfies the following criteria:

1. The use or disclosure of protected health information involves no more than a minimal risk to the privacy of individuals, based on the researcher providing the following:
   a. An adequate plan to protect the identifiers from improper use and disclosure;
   b. An adequate plan to destroy the identifiers at the earliest opportunity consistent with conduct of the research, unless there is a health or research justification for retaining the identifiers or such retention is otherwise required by law; and
   c. Adequate written assurances that the protected health information will not be reused or disclosedto any other person or entity, except as required by law, for authorized oversight of the research study, or for other research for which the use or disclosure of protected health information would be permitted by the Privacy Rule.
2. The research could not practicably be conducted without the waiver or alteration and could not practicably be conducted without access to and use of the protected health information.

Description of the protected health information for which use or access has been determined to be necessary by the Privacy Board:

Subjects: Patients must be children ages 6 months to 18 years.
Patients must be treated for one of the following common acute respiratory tract infections (ARTIs): acute otitis media, group A streptococcal pharyngitis, acute sinusitis, and pneumonia.
Patients must have been seen during the time period of 2026-2029.
We also plan to collect consumer experience data from these patients' parents/guardians who complete exit surveys for these ARTI encounters. This is routinely administered to all patients/families receiving care and are used for a variety of purposes- our group will be looking specifically for comments about antibiotic prescription selection and duration.
Inclusion for parents/guardians:
Parents and/or guardians of children being treated for specified ARTIs.

Date Range: 01/01/2023 TO 01/15/2029
PHI Elements: Name, Medical Record Numbers (e.g. EMPI, EMMI, Encounter Number, Account Number, etc.), Subject Related Dates (e.g. birth date, dates of service, admission date, discharge date, date of death, etc.), ZIP Code

This waiver or alteration of authorization has been reviewed and approved through expedited review. The Privacy Board reviewer has determined the research involves no more than minimal risk to the privacy of the individuals who are the subject of the protected health information.
