# Supplementary material for: Implementation of a pediatric antibiotic stewardship intervention across a large integrated health system: protocol to optimize antibiotic selection and prescription duration for acute respiratory tract infections in children
Source: Implement Sci Commun. 2026 Apr 9;7:93. doi: 10.1186/s43058-026-00915-0 (PMC13181975; doi:10.1186/s43058-026-00915-0)

# Supplementary Tables and Figures

## Figure 3. Intermountain Health System


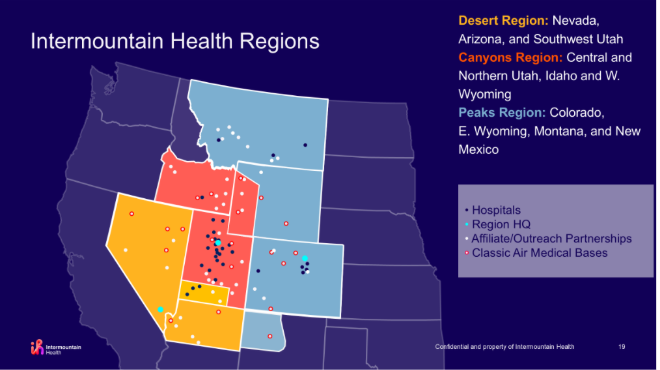


## Table 2. Summary of Antibiotic Stewardship Programs at Intermountain Health

| **Program** | **Region** | **Population** |
| --- | --- | --- |
| **Systemwide Inpatient Antibiotic Stewardship**  **2012 – Current** | Canyons,  Desert, Peaks | 33 hospitals |
| **Adult and Pediatric Urgent Care Stewardship in**  **Treatment of Respiratory Infections (SCORE-UC) 2018 - Current** | Canyons, Desert | 7 KidsCare sites in Canyons  27 Urgent Care sites, Canyons 4 Urgent Care sites in Desert |
| **Systemwide Pediatric Tele-Stewardship (primarily inpatient)**  **2021 - Current** | Canyons, Desert, Peaks | 23 hospitals, 18 smaller hospitals |
| **Primary Care Stewardship-Optimizing Duration of Treatment for UTI, Cellulitis, Acute Otitis Media, Sinusitis and Pneumonia**  **2022 - Current** | Canyons | 69 Family Medicine  22 Pediatrics  22 Internal Medicine |
| **Optimizing Treatment of Acute Otitis Media in Pediatrics in Urgent Cares**  **2023 - Current** | Canyons | 11 Urgent Cares, 5 KidsCare in Utah |

## Table 3. Logic Model for SCORE-Peds Quality Improvement

| **Context** | **Determinants** | **Strategies (ERIC)** | **Outcomes** |
| --- | --- | --- | --- |
| Setting*   - Type (Family Medicine, Pediatrics, Urgent Care, Emergency Department, Telehealth) - Rural vs. Urban - Market   Patient Factors   - Diagnosis - Demographics(age, race/ethnicity, preferred language, SES) - ADI and/or ChOI   Provider Factors   - Type/specialty (MD/DO, APP) - Years in practice | Clinician Knowledge of Evidence-Based Antibiotic Prescribing Recommendations  Clinician Recall of Evidence- Based Antibiotic Prescribing Recommendations at Point of Care  Clinician Knowledge of Antibiotic Prescribing Performance Compared to Peers  Environmental Context and Resources  Clinician Motivation or Self- Efficacy to Change Antibiotic Prescribing Habits | Clinician Education   - Grand rounds - Podcasts - Written info (Care Process Models, Fact Sheets with FAQs)   Remind Clinicians   - EMR-embedded prompts and supports - Commitment Posters   Audit and Provide Feedback   - Standardized dashboard (c-SMART) - Automated email reports - Prompts for supervisor review   Facilitation   - Clinical decision support via EMR modifications (e.g., workflow optimizations via order sets) - Individualized review & problem-   solving, i.e., academic detailing (Boost) | Reach   - N patients receiving adherent care and optimal care   Effectiveness   - Adherence rates (selection, duration)   Adoption   - N providers reaching target adherence & proportion of all providers prescribing - N sites reaching target adherence   Implementation   - Attendance at education events - Feedback on education events - Surveys on use of dashboards - Feedback/individual review sessions   Maintenance   - Continued adherence for at least 6 months post implementation |

Abbreviations: ADI = Area Deprivation Index; ChOI = Child Opportunity Index; MD/DO=Physicians; APP= Advanced Practice Providers; EMR = Electronic Medical Record; c-SMART = cBPI Standardized Measurement and Reporting Tool; ERIC = Expert Recommendations for Implementing Change19

## Table 4. Main project activities by phase

| **Phase** | **Main Project Activities** |
| --- | --- |
| **Pre-Implementation:**  [ 12 ] months Jan – Dec 2025 | **Measure and Report phase**   - Activate Implementation Team - Coordinate with organization team (see Project Team Experience and Environment) - Identify Region/Service Line Champions - Collect and review baseline data on adherence outcomes (see section E. Effectiveness) - Identify best channels for communication, education, and training (i.e., what sources of evidence do providers trust?) - Conduct Family Advisory Council (FAC) meetings, translate findings into plans - Design and test Digital Technology Services (clinical decision support [EMR modifications], c-SMART dashboards) to ensure provider data across all pediatric ambulatory settings for this proposal are captured - Construct education & training protocols - Identify and train Region/Service Line Champions & Site Facilitators - Build best practice packages for routine cBPI quality improvement (main implementation strategies) as well as Boost package (academic detailing) |
| **Implementation**  [ 24 ] months  Wave 1:  Jan 2026 – Dec 2027  Wave 2:  Jan 2027 – Dec 2027 | **Driving High Adherence phase**  Implement cBPI quality improvement sequentially in each region including the following strategies:   - Education (e.g., grand rounds, podcasts, posters, brochures) - Remind & Facilitate (clinical decision support [i.e., EMR modifications]) - Audit and Feedback using c-SMART automated dashboards with comparisons to peers, market, region) - Continual monitoring and adjusting per cBPI Drive High Adherence Protocols; look for differences in adherence by region, community, provider type, subspecialty, patient factors, etc. (see Context in Table 2.) - Implementation Team completes Sustain Assessment and Plan   Explore Boost using Site Facilitators delivering audit and feedback Extend Boost based on findings |
| **Maintenance at Project Sites**  [ 6 ] months Jan – June 2028 | **Sustain phase**   - Continued monitoring of c-SMART dashboard for program effectiveness and sustained adoption - Organization implements items identified by Sustain Assessment and Plan - Publication of findings in scientific journals and submission to appropriate conferences |

## Figure 4. Example Care Process Model and Pocket Card


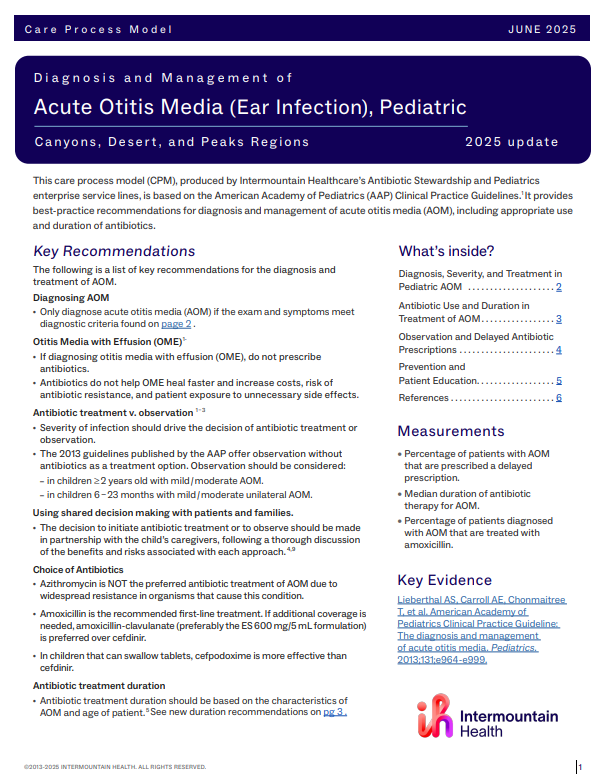


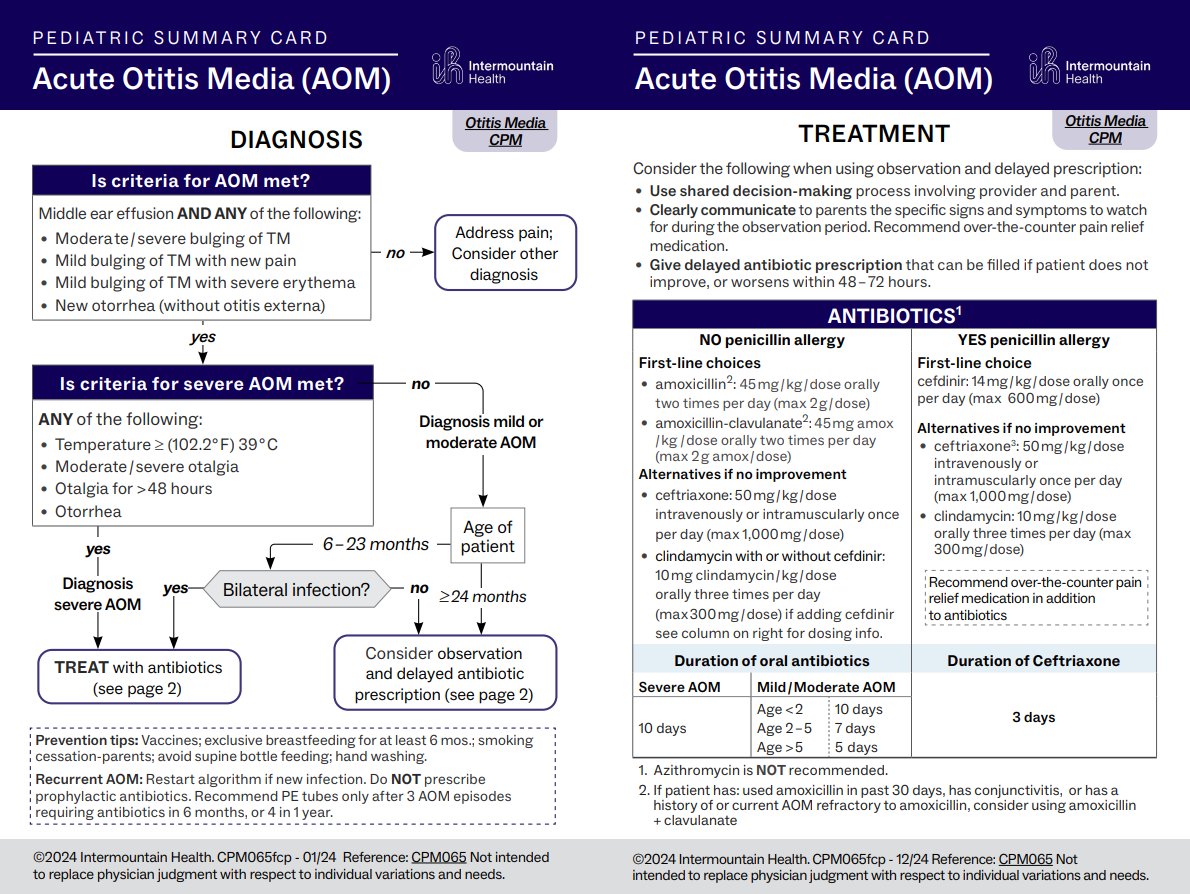


## Figure 5. Specific definition and calculation for Recommended Duration and Adherence Rate (RADAR) metric


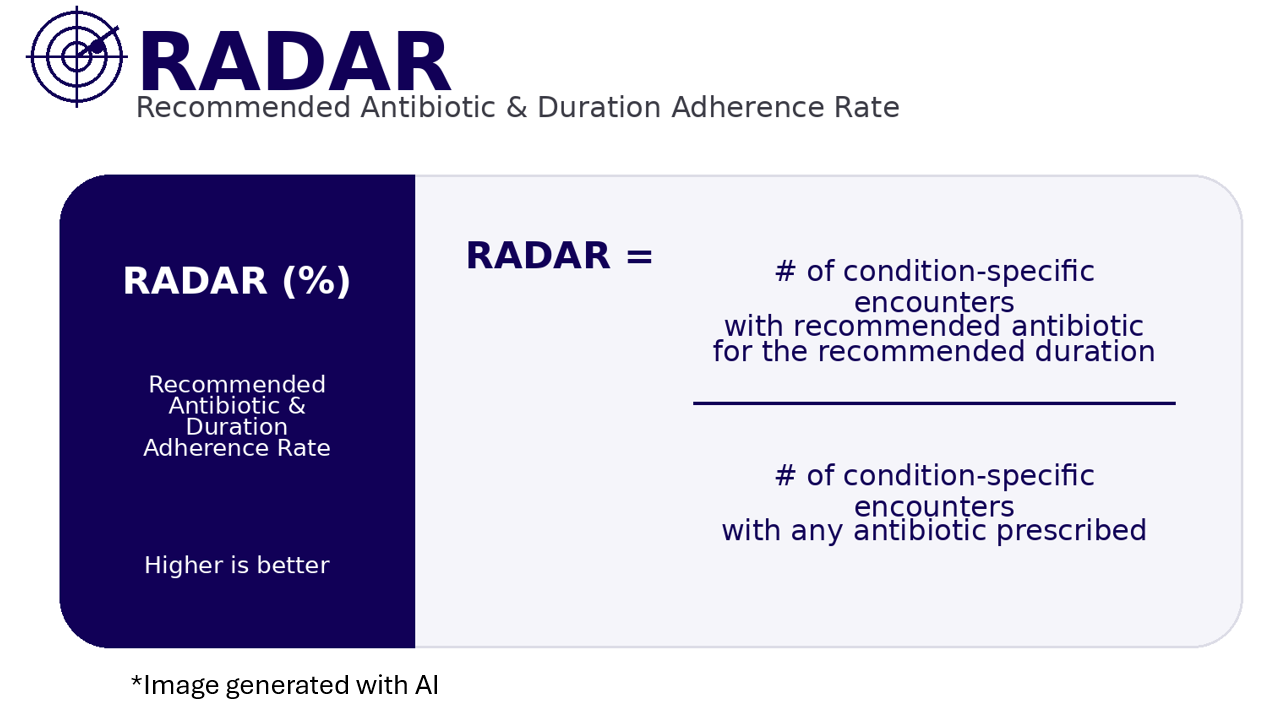

Supplement: Supplementary file 4 — Supplementary Material 4. [file 43058_2026_915_MOESM4_ESM.docx]
